# The Treatment of Pueperal Infection

**Published:** 1907-10

**Authors:** Geo. H. Noble

**Affiliations:** Atlanta, Ga.


### Journal-Record of Medicine

Successor to Atlanta Medical and Surgical Journal, Established 1855, and Southern Medical Record, Established 1870.

OWNED BY THE ATLANTA MEDICAL JOURNAL CO.

Published Monthly.

*Official Organ Fulton County Medical Society, State Examining Board, Presbyterian Hospital, Etc.*

VOL. IX. OCTOBER, 1907. No. 7

BERNARD WOLFF, M. D., EDITOR, 319-320 Prudential Bldg.

GEORGE BROWN, M. D., BUSINESS MANAGER, Business Office 1½-5½ S. Broad St.

E. G. BALLENGER, M. D., ASSOCIATE EDITOR AND ASSISTANT BUSINESS MANAGER.

### ORIGINAL COMMUNICATIONS.

### THE TREATMENT OF PUERPERAL INFECTION.*

By Geo. H. Noble, M. D. Atlanta, Ga.

In considering this subject I will not deal with preventive treatment, as that does not directly concern diseased conditions, but pertains to the management of healthy subjects with a view of preventing disease.

Since there has been a general acceptance of the fact that puerperal infection does not differ from ordinary wound infection, reference to etiology will be limited to two points influencing intelligent application of remedies. First, sapremia, the condition produced by the absorption of proformed ptomanies; second, septisemia, or septic infection, the entrance into the circulation of various forms of infecting micro-organisms.

In sapremia, putrefactive changes in secundines or blood clots, remaining in the cavity of the uterus are the causes of the poisons absorbed. I trust, therefore, you will pardon the mention of

*NOTE.—Since the above was written I have had several more labors in contracted pelvis to conduct. Of these the most interesting was that of B. H., aet. 37, iv para. Her first two children were girls, delivered p. v. by forceps. The third was a boy, delivered by Cesarean section after failure of attempts at forceps delivery.

†Read before the Washington Obstetrical and Gynecological Society, Feb. 15, 1907, and reprinted through the courtesy of the American Journal of Obstetrics.

some conditions predisposing to incomplete evacuation of the uterus. In many cases it is due to hastened delivery of the after-birth by expression and traction upon the cord before hte placenta has separated from the uterus. This has a tendency to produce marked tonic spasm of the womb, a condition recognised by a prolonged and marked contraction that reduces the uterus much below its normal size. Continuation of such an abnormal strain upon the muscles of the uterus results in exhaustion and relaxation, with slight hemorrhage in the cavity. On the coagulation of the blood the uterus is stimulated to renewed contraction, but with insufficient force to expel the clots. The uterus again relaxes and more or less inertia follows, renewed hemorrhage in the cavity of the uterus takes place, and the clots increase in size. This is repeated from time to time, the uterus growing larger as it fills with blood. The woman becomes more and more exhausted, and the uterus tired from overwork, so that there is finally complete inertia. Then one of two things follows, either a severe hemorrhage or retention of the clots, and later sapremia. Smaller clots may be retained in tonic contractions of the uterus when one or more of the uterine sinuses are not completely freed from placental tissue, a condition unfavorable to the formation of coagula in the ends of the veins.

Another cause for retained portions of placenta is deformity of the womb. While this occurs only in a small percentage of cases, I think it is encountered more often than is geneally presumed. I refer to the bicornate uterus, and especially to slight depressions in one or the other angles of the uterus. These are often so slight that the unevenness of the fundus may not be detected by the hand grasping it through the abdominal walls. Yet there may be pockets from one to two inches in diameter, more or less hemispherical in shape. Sometimes the plugs of placenta removed from them will look like half a baseball, or other spherical objects of smaller sizes.

It is important in examining the uterus to notice carefully the degree of firmness exhibited at various points on the inner surface of the womb, for depressions may be so filled with placental tisue that the surface appears to lie in a continuous plain with the other parts of the uterine mucosa. But pressure with the end of the finger will detect a boggy sensation characteristic of placental tissue. It will also be noticed that during contraction the uterine tissue of the placental site becomes firm and resisting while plugs of placental tissue in pocket deformities have a tendency to bulge into the uterine cavity and are but slightly increased in tension or density. The placental site may also be distinguished from such masses by the interlobular bands that form trabeculae over its surface. They resist considerable pressure by the finger, while the spaces between them are soft and spongy in the intervals between contractions.

Again I have seen in the bicornate womb where the tubo-uterine opening much relaxed, portions of the placenta extending into the Fallopian tube.

In the uterus bicornis unicollis with the fetus in one cavity and the afterbirth in the other, I saw a case in which that portion of the amniotic sack filling the fetal side was left intact. The true condition was not suspected because the sack of the placental side was apparently large enough to contain both fetus and afterbirth. In this instance the attending physician did not recognize the double character of the womb.

While sapremia is not usually marked until the third day, or after, it may prove fatal in thirty-six or forty-eight hours where large quantities of toxins are rapidly absorbed. I have observed this in twenty-four hours retention of seperated placenta and membranes The odor accompanying such decomposition was not present and toxins were not as virulent as they might have been a few days later, but the enormous quantities absorbed were sufficient to saturate the patient.

The treatment consists in complete removal of all debris found in the cavity of the uterus as soon as the first chill has subsided. This is best done with the finger, sweeping its palmer surface over the entire surface of the cavity of the uterus, breaking into a pulp all particles that are too large to be easily removed, and then washing them out with a brisk stream of sterile water. Pieces adhering closely to the placental site that cannot be removed by side to side motion of the finger, may be dislodged by the finger nail, forcing it from below upwards in the long axis of the uterus. In like manner shreds of membrane may be detached, so that the use of the curette is rarely required, except to hook out large, loose masses, and even then lithotomy forceps, shaped after the pattern of obstetrical forceps, will be found much more serviceable.

If the finger does not readily reach the fundus, the entire hand should be carried into the vagina and the uterus telescoped down upon the examining finger by supropubic pressure.

In septicemia, the infection enters the circulation either through the lymphatics or blood-vessels. It may be mild or fatal, according to the intensity or degree of invasion. Mild cases are usually confined to superficial invasion of the tissues of the uterus, but grow more serious as extension into deeper structures occurs, resulting in septic softening of uterus, abscesses, peritonitis, thrombophlebitis, or pyemia. Extension takes place through the lymphatics and bloodvessels, and less frequently along the mucosa to the Fallopian tubes in which septic foci may form.

The infecting agent is most frequently the streptococcus or the staphylococcus, and less frequently the gonococcus, the colon, or diphtheria bacillus, gas producing bacilli, etc.

Superficial invasion may become extremely serious when sufficiently intense to cause necrosis and sloughing of tissue. There is then a mixed toxemia; septicemia plus sapremia.

The treaatment of septicemia is systemic and surgical. In both the vital forces should be fostered.

By systemic treatment I refer to remedies that are intended to combat the disease through the agency of the circulation.

The two principal agents are Crede's collargol, or other silver preparation, and antistreptococcus serum.

Collargol is supposed to act as a systemic antiseptic. It is used by inunction, hypodermic, and intravenous injections. It has a few adherents in Europe, and they claim where it fails that it is because insufficient dosage is employed. This argument is hardly logical, for some cases will recover without treatment, and others are hopeless at an early stage. Collargol has excited no euthusiasm in America, and I have no knowledge of its value.

Antistreptococcus has not sustained the claims made for it. This is shown by the report of a committee appointed by the American Gynecological Society in 1899 to investigate the subject. Their report in substance was that it has no curative effect on puerperal infection. The same conclusions are reached by all individuals that I have been able to consult. Whitridge Williams says it may be prophylactic, but is not a curative remedy when used after the process of infection is well established, and that it is not an antitoxine, but acts by stimulating leucocytosis. At best its application is limited, for it is offered for use in pure streptococcic infection only. Certainly the serum cannot accomplish results in advanced thrombophlebitis or peritonitis. My testimony is unfavorable to it.

Disappointed then in the methods of systemic treatment, we are forced to rely upon surgical relief. For an intelligent application of remedies, an accurate diagnosis is necessary, not so much as to the character of infection, even though it is desirable to know the kind of infection we have to deal with, but as to the tissues involved, the extent of invasion, and location of infected centers.

For instance, mild cases are usually confined to superficial invasion, or practically to the uterine cavity, the mucosa, and immediately underlying tissues. Deep invasions may involve the uterine muscularis, uterine ligaments, peritoneum, pelvic veins and lymphatics, tubes, ovaries. Less virulent infection may involve the deeper tissues with minor constitutional impression than intense toxicity of limited penetration. Therefore, it is pertinent to consider the intensity of infection as well as the extent of invasion.

ANTEPARTUM INFECTION.

Septic infection may be antepartum or postpartum. The antepartum form has its origin usually in separations of the placenta, resulting in hemorrhage and accumulation of clots, forming a fertile culture medium. Mild cases frequently recover after evacuation of the uterus with proper precaution, but otherwise they should be treated as superficial postpartum invasions.

Where the process is extensive and a large surface of the placental side is involved, the condition becomes extremely serious, Labor is slow because uterine contractions are inefficient. This is due to septic softening, rendering the muscularis involved incapable of contracting. The remaining portion of the uterus becomes exhausted, more or less inertia follows, and instrumental delivery is often required.

If there is much involvement of the deeper uterine tissues, the womb fails to contract after delivery, and form the usual hard globular mass found behind the pubic bone. On the contrary, it is soft and flabby, and feels like an empty bag lying in the hollow of the sacrum and extending above the brim of the pelvis. Its outlines are difficult to determine. The discharges collect in the relaxed uterus, and infection extends to other parts of the organ. Constitutional symptoms are pronounced and the patient rapidly grows worse. With streptococcus infection, the production of pus may be copious, and if the process extends to the peritoneum, marked depression with low temperature and rapidly increasing pulse and varied delirium are noticeable symptoms. The condition is grave and demands heroic measures, as treatment to the cavity of the uterus is ineffectual from accelerating drainage.

Hysterectomy, therefore, is demanded in the first few days after delivery, in face of the warning that this form of infection is liable to excite peritonitis by contamination during the operation. In the class of cases under consideration, peritonitis is present in a very violent form, and a few hours delay may mean the death of the patient, for they usually terminate fatally in the first week. Hysterectomy to be of any service must be done promptly, even where diagnosis of peritonitis is doubtful, for the liability to occurrence and extension of thrombophlebitis into the pelvic veins is very great. When hysterectomy is delayed until this takes place, either pyemia supervenes or the removal of thrombosed veins adds to the magnitude of the operation.

I am so convinced of the necessity of early surgical interference in advanced antepartum sepsis that I do not hesitate to recommend its prompt execution. Delay increases mortality and discredits the operation.

POSTPARTUM INFECTION.

The milder form of postpartum infection, when practically confined to the cavity of the uterus, yields readily to the Carosa treatment, but, in the advanced process, involving deeper tissues of the uterus, this is useless. It depends upon three principles for its effectiveness: First, promotion of drainage and the prevention of the accumulation of discharges in the cavity of the uterus by filling the latter with gauze; second, the antiseptic effect of the alcohol used; third the dehydrating and hardening of the epithelium and other superficial tissues, which to a certain extent prevent absorption of septic matter.

While little confidence can be placed in the antiseptic action of irritating or coagulating fluids, such as strong solutions of bichloride of mercury and carbolic acid, alcohol is serviceable. The treatment consists, first, in a thorough cleansing of the vagina and uterine cavity; second, introduction into the uterus of a small sterile catheter, to the tip if which is stitched a narrow strip of sterile gauze. The latter is loosely packed around the catheter until the cavity is filled (without pressure). The cervical canal is not packed, but a piece of the tape is left projecting in the vagina to facilitate removal. The end of the catheter is then brought out through a sterile dressing, and protected by a second dressing, the removal of which renders access to the catheter easy without interfering with the pads next to the vulva. Through the catheter one or two drachms of fifty or seventy-five per cent. alcohol is injected every twenty or thirty minutes for the first twenty-four or forty-eight hours, and as the temperature declines the intervals between injections are gradually lengthened. The dressing is left in place from four or five days to a week. At the end of that time the infection is under good control. But, if the temperature shows a disposition to rise, the treatment is again resumed.

If, with careful application of this treatment, the temperature and pulse continue to increase, it is evident that the process of infection has extended beyond the uterine cavity and that local treatment is useless. ,

Irrigation of the uterus is often abused by a lack of discretion in its use, and when admissible may be harmful from carelessness or rough handling of the syringe nozzle by abrading the mucosa or breaking through the exudate underlying the infected area. In this way atria for renewed absorption are formed. This is liable to occur in the use of metallic or other hard instruments. To be effectual, a bold stream of sterile water should be used at short intervals, the time being so regulated that the patient's temperature is not allowed to rise. It acts mechanically and can accomplish no good in deep seated infection, therefore its usefulness is confined to superficial invasions when the mucosa is covered with necrotic or sloughing tissue. This condition is recognised by the escape of dirty looking debris, with perhaps some offensive discharge, patulous os uteri, and the absense of the marked flabby condition of extensive invasion of the muscularis uteri. Little or no interference with involution of the uterus (firm uterus, contracted os, healthy discharges) indicates that the uterine cavity is practically free, that the infection is deep seated, and the use of douche is contraindicated. When irrigation does not control the fever, we know the treatment is misdirected.

At best flushing is neither as safe nor as reliable as the Carosa treatment, and is more troublesome and annoying to the physician and patient.

When infection involves deeper structures, the process may follow two or three courses: (A) If in the direction of the peritoneum, peritonitis may result, (B) but when the infection becomes more or less sterile, it may be localized in the walls of the uterus forming delayed abscesses. (C) When it invades the sinuses in the placental site thrombophelbitis to a greater or less degree results.

Peritonitis usually occurs in the more violent forms of infection, because in milder cases hematic osponisis modifies the process, consequently the symptoms develop in a few days, whereas circumscribed abscesses are not detected until the second or third week, er later. In puerperal peritonitis prompt action is necessary. A few cases do recover under simple abdominal section and drainage, others under vaginal incision and iodoform gauze packing; but the latter treatment has an exceedingly limited field of application, because of its indirectness in reaching the septic centers. If the peritonitis is confined to the posterior walls of the uterus, gauze packing may excite adhesive inflammation and pouring out of enough exudate into and beneath the peritoneum to block the lymphatics, but its application requires exactness of diagnosis, so that its practical employment is narrowed down to the drainage of pus collections and free fluids in the pelvis.

Hysterectomy in peritonitis is unpromising, upon one hand, from the fact that it is delayed too long, resulting in high mortality. While upon the other hand, early operations are attended with less mortality, we recognize a liability to an unnecessary sacrifice of the uterus in some cases. It is hard, therefore, to draw a line of distinction, and where one has the courage of his conviction, it is almost impossible to convince the patient and her friends that the operation is needful.

Abscesses occur at the end of the second week or a little later, after the most acute symptoms of sepsis have passed. The cavity of the uterus, therefore, is apparently free from serious infection or debris. The temperature curve is septic and devoid of chills, unless complicated with thrombophlebitis.

If the pelvic cavity is not obstructed with exudates, these abscesses may be felt through thin or relaxed abdominal walls. When the abscess is single a rounded, slightly flattened outline may be felt on the peritoneal surface of the uterus, which, to the sense of touch, feels something like a small, deeply seated fibroid tumor, but is more elastic, and the outlines are not so sharply marked. When the Fallopion tubes and cavity of the uterus are not infected and the uterus is firm in texture, diagnosis is not likely to be confused with other conditions, except thrombophlebitis. The latter is marked by chills, sharp rise in temperature, and rapid decline to normal. This is repeated once or twice in twenty-four hours. In the interval the patient's conditions is apparently good (except late in the disease.) In the late cases frequent pulse, pyemic temperature curve and generally bad condition are noticeable. The absence of hard, cord-like ridges along the course of the veins is a valuable sign in excluding this complication.

I have collected nineteen cases, all of which have been verified either by operation or autopsy. Of this number four were post-mortem findings, four hysterectomies, with one death, and eleven others that were incised and drained with eleven recoveries. The location of the abscess was mainly in the region of the fundus, or at points quite accessible through abdominal incisions. They were either sub-peritoneal or just under the outer muscular layer of the uterus. This is accounted for, perhaps, by the fact that the main blood supply to the uterus is through the layer of muscle, consequently the vascularity of the parts favored phagocytic action at this point, and the spread of infection becoming modified, suppuration resulted at the place where the barrier appeared. If such is not the case suppurating centers should be found in deeper parts or the uterine walls with equal frequency.

In one case there was thrombophlebitis of the pampinaform plexus, uterine and ovarian veins of the right side. The condition was such the veins required ligation and excision as high as the brim of the pelvis. The case had four retention abscesses between the coils of the intestines. Another had five abscesses similarly situated. In the latter a prominent surgeon tried to drain through the vagina, making two attempts, but failed. When I opened the abdomen, the utter hopelessness of drainage by that route was apparent. The pus centers were too high in the abdomen and the fundal abscess so inaccessible that the fingers could never have reached it from below or, if perchance the uterine abscess could have been reached, it very likely would not have been reached, it very likely would not have been detected, as its elevation above the surrounding surface was so limited. These abscesses can be better handled through an abdominal incision, even if it should be desirable to drain below.

The expectant plan of treatment promises nothing. They all die. The only means of relief, therefore, is through surgical intervention.

Hysterectomy has given a mortality of twenty-five per cent., which perhaps would have been greater if a larger number of cases had been treated. All drainage cases recovered; eleven in number, eight in my hands, three in others. This is good showing when compared with the high mortality of puerperal hysterectomy.

It is but a reasonable conclusion to say that an abscess of the uterus in the puerpal woman should be treated as similar pus collections elsewhere; that is, on general surgical principles; exceptions however, should be made in cases of multiple abscesses where the branches of successful drainage are doubtful. Besides, the uterine musculature is so much involved in septic inflammation nothing short of hysterectomy will answer.

As thrombus of the pelvic veins occurs in six or seven per cent. of puerperal infection with high mortality, considerable saving of life may be made by early recognition of the disease and prompt interference. It may begin early, but usually the symptoms are not clearly defined until late.* The infection follows the uterine, ovarian, round ligament, and hypogastric veins, and the coagulations may extend far enough to block the inferior vena cava.

The disease is often complicated with septic conditions of other parts, such as involvement of tubes ovaries, suppuration in the parenchyma of the uterus, parauterine tissues; or the clot itself may be permeated with pus, the latter escaping directly into the circulation.

The size of the trombus does not always correspond to the gravity of the symptoms. Intense infection of a small thrombus may cause more marked symptoms than a large clot of less virulence. This was illustrated in a case of mine at the Grady Hospital. The clot was confined to the vein of the right round ligament and was only three-sixteenths of an inch in diameter and two inches in length. The symptoms were violent, but were immediately and completely relieved by removal of the vein. Upon the other hand, in another case with involvement of the pampinaform plexus, uterine and ovarian veins of the right side, the symptoms did not differ materially from a mild case of puerperal infection of the uterine mucosa.

*Exceptionally the symptoms may appear earlier. In Blot's Case (A. J. C. Med., 1905, p 368). the chill came on the third day. and on the ninth the thrombus had advanced to such an extent resection of the left side of the uterus and the corresponding broad ligament was required.

The symptoms, according to Heiderman, are headache, slight irregular rise in temperature, small, frequent pulse (gradually but steadily increasing,) dysuria, and ischuria, sudden pain in the side, pains in bending hip (in femoral vein under Pourpart's ligament,) lassitude, and vertigo on leaving bed. Howard* says "temperature and pulse rise rapidly, rigors supervene, followed by profuse sweating; tongue is dry; appetite is lost, and there may be delirium. To these symptoms are soon added those of pyemia; rapid oscillations of temperature, rigors, sweating, and dissemination suppurations." Mohler's symptoms is gradually and steadily increasing pulse (climbing.) In bad cases it is a most important one. Vany states that the pulse and temperature curve vary. They may go up in successive bounds as the temperature comes down. Each forward leap of the pulse curve corresponds to a new extension of the clot.

In all cases reported, the chill is a prominent feature. It usually comes on in the second or third week, showing a tendency to slow extension and pathogenic invasion of clot, but may be deferred, as in one of Duret's cases, to the fortieth day, or perhaps later. If the external or common iliac vein is involved, there will be swelling of the leg, and the duration of the disease may be prolonged as much as seventy-four days or more. But there are cases of very short duration, which are sometimes marked by complication, and may be entirely overlooked on account of the ab-cases of apparently mild type may have great incolvement ot veins sence of suggestive signs and symptoms.

While the above symptoms are commonly met with in the disease, they represent cases that are influenced more or less by the process of suppuration, and, therefore, differ materially from those that characterize uncomplicated and non-suppurating, but infected thrombi. I have observed that the latter give the following chain of symptoms.

The disease may or may not be preceded by signs and symptoms of slight septic disturbances for the first week or two. These manifestations, whenever present, become less marked and may entirely disappear, leaving the patient apparently convalescent. In the second or third week the first pronounced symptom is the chill, which is well marked if infection is intense. This is accompanied by rapid pulse and sharp rise of temperature to 103 to 105 degrees terminating in rather rapid decline to or near normal. These symptoms usually occur once in twenty-four hours, but may be repeated in shorter intervals of time. In the interval the patient is apparently in a normal condition, except that she is left weaker after each attack. The change may be slight, consequently one may be deceived into the belief that each chill will be the last before convalescence begins, but, on the contrary, the pulse shows gradual loss in tension and volume and an increase in rapidity (climbing.)

The pelvis may be negative on examination if the thrombus is small, but when large or late in the disease the veins feel like tortuous cords to the fingers.

When the infection extends through the walls of the vessels, the paravascular tissue may be hard and nodular, the general direction of the exudate taking the course of the vessels, which is a material aid in diagnosis. Such exudates are not markedly sensitive or painful to pressure in bimanual examinations.

Diagnosis of uncomplicated puerperal thrombosis of the pelvic veins is made by taking into consideration the above symptoms with the results of careful pelvic examinations. The value of the latter consists mainly in the ability to exclude other conditions. If in the examination of the pelvis, the process of involution of the uterus appears not to have been materially interfered with; that is, if the uterus is firm and reasonably small for the period of puerperium, if the cervival canal is not patulous, and no unhealthy discharge is escaping from it, it is reasonable to suppose that the uterus is not involved. Serious infection of the uterus may then be excluded, and the same may be said of the Fallopian tubes and ovaries when they are not palpable or buried in exudate.

Thrombosis due to laceration of the vagina is comparatively rare. This source of origin, therefore, can usually be disregarded, especially if the lesion is slight in extent; but if the tear extends high up in the vagina or deep into the ischiorectal fossa, the lateral vaginal plexus of veins may become involved. When this occurs they are covered up at an early stage of the disease by exudate, and in the place of cord-like vessels being felt on the side of the vagina, a nodulous ridge may lead up to the base of the broad ligament. When this conditions is found, if other parts of the pelvis are free, localization of the thrombus in the lateral vaginal veins can be safely determined.

In differentiating between thrombosis and intramural abscess of the uterus, uncomplicated by peritonitis and adhesions, in the latter small rounded flat elevations upon the surface of the uterus may be felt in patients with very thin or relaxed abdominal walls. Such cases present the ordinary septic pulse and temperature curves, and do not have the distinct interval of non-suppurating thrombus. When complicated by adhesions, or diseases of the appendage with tense abdominal walls, exclusion of intramural abscess and detection of tortuous veins are extremely difficult or impossible.

Suppurating thrombosis will very likely be overlooked when complicated with peritonitis, or pus tubes, unless symptoms of pyemia supervene, or the pulse shows a tendency to become climbing in character.

The treatment is mainly surgical* for when a diagnosis is made most cases are well advanced into the third or fourth week, which is ample time to test temporizing treatment, and mild cases either show signs of improvement earlier or go unrecognized altogether.

Any puerperal case with pelvic lesions, variable temperature, and climbing pulse of three to four weeks' duration without signs of improvement justifies an operation of some kind, especially if the uterus proves negative as the source of trouble. If a mistake is made in diagnosis, and the locus infectus is found in the Fallopian tubes, or abscesses in other parts of the pelvis, no surgical error is committed, for they too are in need of serious attention.

There are several plans of procedure: First, excision of thrombosed veins; second, ligation of thrombosed veins; third, hysterectomy. In addition to these, attention to the complicating conditions must be given.

Some bad cases with small but intensely infected clots may be quickly relieved by removal of small veins. Upon the other hand, cases of apparently mild type may have great involvement of veins requiring extensive excision, therefore the magnitude of this operation cannot be determined beforehand. Prolonged cases with extensive involvement requiring trying operations and are less able to stand them.

Guicciardi's objection to excision of veins is the liability of reinfecting the wound by soiling the parts with clot. How great the liability to this accident is I cannot say, but in two of my cases in which the veins were lacerated by digging the clots out of the brood ligament with the fingers, the temperature range for two or

---

*In this I do not include all cases of phlegmasia alba dolens, for often this does not depend upon local (or pelvic) lesions, but upon other causes.

three days was quite irregular, and reached high marks at short intervals (103 to 105 deg.,) but some of this temperature may have been due to the extensive surface involved. Each of these had numerous abscesses in the uterus and ovaries, and pus collections between the coils of the intestine, besides the omentum and intestin were softened and adherent from the umbilicus downward. As the chill was not repeated in either case, it is very likely that soiling the wound with clots had little to do with it. This may be avoided, however, by double ligation and cutting between to prevent coagula escaping from the ends of the vessels. Should the vessel be lacerated, as in the cases mentioned, a drain may be inserted to protect the peritoneum.

A very few cases have been reported. The number of excisions in Guicciardi's collection is too small for just conclusions to be drawn and the conditions may vary so much that the methods may not have had equal advantages. Even the addition of five cases (one by Boldt and four of my own) so modified the mortality that the showing for this method of operating quite surpasses ligation. It is theoretically the most surgical, but it must have its limis, particularly in cases that cannot stand long operations. In early cases this method is to be preferred, especially if the clot is confined to the veins of the true pelvis.

Ligation of the veins is not satisfactory, especially if the clots become infested with pyogenic cocci. Pus in a large vein shut out from the circulation by a ligature only is a hazardous condition. If the ligature is of soluble material, it may disappear or loosen too soon and let a flood of pus into the general circulation. Neither is it a sufficient barrier to the further progress of infecting micro-organisms. The paravascular infection may pass beyond the obstructing ligature and by permeating the walls of the vessels infect the new clot on the proximate side and renew the process of thrombophlebitis.

Hysterectomy in this condition is extremely dangerous. In the first place, it is often delayed too long to successfully remove all the infected centers. When thrombosed vessels are confined to the uterus it has some chances of success, but propagation through the vessels into the broad ligament extends the infected field into the pelvic veins, and at other times the uterus may not be at fault, for the thrombi may lie exclusively in vessels outside of that organ. Removal of the uterus in such circumstances is worse than useless, as it would leave the thrombosed vessels undisturbed.

Upon the other hand, excision of the diseased veins without hysterectomy when the uterus is not involved is promising, except in extreme cases.

In the summary of the small number of cases accessible the results are as follows:

| | |
|---|---|
| Resection of veins, mortality | 28 4-7 per cent. |
| Ligation of veins, mortality | 44 4-9 per cent. |
| Hysterectomy, mortality | 64 1-4 per cent. |

From this I venture the assertion that until further experience shall have worked out the solution of this problem, we must accept the opinion that early recognition of septic thrombosis of the pelvic veins and prompt excision is the best method of surgical relief we can offer our patients in this complication of the disease.

There is a strong point in the treatment of puerperal infection I wish to make, that is, nearly all of these cases are either mild in character or of superficial invasion in the beginning, so that if proper treatment is employed at such times the progress of the disease will be modified and the mortality greatly reduced.

